# Changes in stroke risk by freedom-from-stroke time in simulated populations with atrial fibrillation: Freedom-from-event effect when event itself is a risk factor

**DOI:** 10.1371/journal.pone.0194307

**Published:** 2018-03-12

**Authors:** Tomoki Nakamizo, Masahiro Yamamoto, Ken Johkura

**Affiliations:** Department of Neurology, Yokohama Brain and Spine Center, Yokohama, Kanagawa, Japan; Maastricht University Medical Center, NETHERLANDS

## Abstract

The risk of atrial fibrillation (AF)-related stroke is usually assessed by calculating the CHA_2_DS_2_-VASc score, the components of which are various risk factors, including prior stroke. Although prior stroke is considered the strongest risk factor, the associated risk is actually inferred. Nevertheless, it implies a “freedom-from-event effect” (FEE)—the longer a patient is stroke-free, the lower the stroke risk. Although dynamic prognostication has been applied to cancer, the FEE has been ignored in AF, probably because of methodological difficulties. We conducted a simulation study to evaluate the FEE in the risk of AF-related stroke. We modeled various populations of AF patients and simulated the development of stroke assuming a nonhomogeneous Poisson process, where the hazard depends on age, comorbidities, and individual variability. Parameters were set so that the model respects the CHA_2_DS_2_-VASc scoring scheme and reproduces the 1-year CHA_2_DS_2_-VASc score-wise stroke risk and relative risk conferred by real-world risk factors. We tracked stroke risk over 0 to 15 years of freedom-from-stroke time (FST), both prospective FST (pFST), which begins at the time of diagnosis and continues to the future, and retrospective FST (rFST), which begins at the present and looks backward to the time of diagnosis. The pFST counterbalanced the increase in stroke risk conferred by aging; in patients with a CHA_2_DS_2_-VASc score of 1, the pFST offset 62% of the age-conferred risk increase. The rFST reduced the stroke risk; in patients with a CHA_2_DS_2_-VASc score of 2 and without prior stroke, an rFST of 6.8 years reduced the stroke risk to the midpoint between CHA_2_DS_2_-VASc scores 1 and 2. The study results suggest that the FEE should be considered in evaluating stroke risk in patients with AF. The FEE may be important in other recurrent diseases for which a prior event is a risk factor for a future event.

## Introduction

Cardioembolic stroke attributable to atrial fibrillation (AF) results in morbidity and mortality greater than those resulting from non-AF related stroke [1], and the stroke-related costs are higher [2]. Moreover, the prevalence of AF-related stroke is increasing, exacerbating the clinical and economic burden worldwide [3,4]. A patient’s risk of AF-related stroke and of thromboembolism elsewhere in the vascular system can be greatly reduced by oral anticoagulation (OAC) with warfarin [5] or a direct oral anticoagulant [6]. However, because OAC therapy poses a risk of fatal hemorrhage [7], it should be initiated only when the benefit outweighs the risk [8,9]. Thus, before starting OAC therapy in a patient with AF, the physician should evaluate the patient’s stroke risk by noting the presence of risk factors, such as advanced age, any comorbidity (hypertension and/or diabetes mellitus, for example), and prior stroke [10,11]. The CHA_2_DS_2_-VASc scoring system [12], which incorporates and scores important risk factors, is used widely for clinical risk stratification.

Of the risk factors included in the CHA_2_DS_2_-VASc score, prior stroke is deemed the strongest [10,11]. However, it is not biologically possible for a stroke to induce a future stroke. Rather, prior stroke is a factor upon which a high stroke risk is inferred simply because stroke has occurred in the patient. When prior stroke is used as a predictor of stroke, no direct biological rationale is present; the rationale is“since they have developed stroke, they should have some underlying causes of developing stroke.” This line of reasoning implies that the longer a patient is stroke-free, the lower the patient’s risk of stroke. If such a “freedom-from-event effect” (FEE) is clinically meaningful, physicians should take the length of time the patient is free from stroke, i.e., the freedom-from-stroke time (FST), into consideration when evaluating the risk of stroke in a patient with AF.

When considering FST, two questions arise. The first is *How does the risk of stroke change as the patient with AF ages without suffering a stroke*? In other words, *When a man diagnosed with AF at the age of 65 has reached age 75 without experiencing a stroke*, *should OAC be initiated or withheld*? In such a scenario, the risk of stroke would be evaluated on the basis of a relation between the FEE and the patient’s age. The second question is *How different are the stroke risks of patients of the same sex and age and with the same comorbidities but with different FSTs*? In other words, *Is a 75-year-old man who has been stroke-free for 10 years after a diagnosis of AF at a lower risk of stroke than a 75-year-old man who has been recently diagnosed with AF and thus not judged to be in need of OAC therapy*? In this scenario, the difference in stroke risk between these patients would be determined solely by the FEE. Hence, there are two types of FST, with the first being what we call prospective FST (pFST), which begins at the time of the patient’s diagnosis and continues as the patient grows older, and the second being what we call retrospective FST (rFST), which begins at the present and looks backward to the time of the patient’s diagnosis.

pFST and rFST are dynamic variables that change over time, and the inclusion of such dynamic variables for prognostication of “conditional survival,” the probability of surviving another *t* years being conditional upon already surviving *s* years, has been shown to add reliability to cancer survival forecasts [13]. In patients with AF, however, the issue of FEE has long been ignored, presumably because of methodological difficulties. Evaluating the FEE is much more difficult than evaluating conditional survival in the context of cancer [13] because in patients with AF, the relation between FST and stroke risk is complicated not only by advancing age but also by the presence of other risk factors. Consequently, there have been no reported investigations into stroke risk as it is affected by FST.

When direct study is not feasible, model-based simulation is useful [14]. Using Bayesian modeling, we previously calculated the relation between pFST and stroke risk, and the model predicted that the FEE will outweigh or partially offset aging, depending on the initial stroke risk [15]. The model was, however, based on theoretical mean values and not a simulation that reflected real populations. In the study described herein, we formulated a real population-based model for the development of stroke in AF patients of various ages and with various numbers of comorbidities. Using this model, we evaluated changes in stroke risk in relation to pFST and rFST in patients of various risk statuses, especially in relation to CHA_2_DS_2_-VASc scores.

## Methods

### Defining “stroke” operationally

With stroke being the focus of the study, we first defined “stroke” inclusively as true AF-related stroke as well as the following AF-related thromboembolisms or events: systemic embolism, pulmonary embolism, and transient ischemic attack (TIA). Such disorders make up a minor portion of AF-related events [12,16–18] and represent less of a clinical burden [3], but they have been included as outcomes in some studies [12,16,17], including those we referred to when we constructed our model [12,16]; they are also included in the risk factor “prior stroke” in the CHA_2_DS_2_-VASc scoring system [12].

### Developing the model

We modeled populations of AF patients of various ages and with various numbers of comorbid risk factors, and we simulated the development of stroke in these populations. The development of stroke was assumed to follow a nonhomogeneous Poisson process in which the hazard depends on the variables used in the CHA_2_DS_2_-VASc scoring system [12].

We introduced into the model four comorbidities: hypertension, diabetes mellitus, congestive heart failure, and vascular disease [10–12]. In addition, we included female sex, another risk factor [11,12,19,20], as a “comorbidity.” Zero to five comorbidities were assigned to the simulated patients.

We also introduced age into the model so that the hazard would increase with age [10–12]. In addition, we assigned to each patient an initial age such that the total patients ranged in age from 55 to 85 years at the time of the diagnosis of AF.

We introduced individual variability into stroke risk unaccounted for by known risk factors. This we achieved by generating a set of normally distributed random numbers and then assigning a number to each simulated patient. We know the risk to vary, even among patients of the same sex and age and with the same comorbidities; hence prior stroke stands as a predictor of future stroke. The random number assignment was done to account for the variability. In addition, the variability was expected to produce an FEE through attrition of high-risk individuals over time, although we did not attempt to model the FEE explicitly.

Thus, the hazard was the function of age, comorbidities, and individual variability. We equipped the hazard function with a linear predictor such that the hazard increased multiplicatively with these variables. However, because the risk in the real world tends to “saturate” as the risk increases [12,16,17,21], we introduced negative quadratic interaction terms such that the hazard saturates when risk factors accumulate. The hazard was made constant beyond the point at which it saturates.

Thus, the hazard function *λ*_*i*_(*t*) of patient *i* at time *t* is

$$\lambda_{i}(t)=\left\{ \begin{array}{l} \;\lambda_{0}\exp\left(\mu_{i}(t)-\kappa {\mu_{i}(t)}^{2}\right),\;(t<T_{i})\\ \lambda_{0}\exp\left(\mu_{i}(T_{i})-\kappa {\mu_{i}(T_{i})}^{2}\right),\;(t\geq T_{i}) \end{array}\right.$$

where

$$\mu_{i}(t)=\sum_{k=1}^{5}\beta_{k}x_{i}^{\left(k\right)}+r(t+\tau_{i}-70)+d_{i},$$

and

$$T_{i}=\{1-2\kappa [\sum_{k=1}^{5}\beta_{k}x_{i}^{\left(k\right)}+r(\tau_{i}-70)+d_{i}]\}/2\kappa r.$$

Here, *λ*_0_ denotes the baseline hazard, *β*_*k*_ is a coefficient that denotes the effect of comorbid risk factor *k* (*k* = 1, …, 5), and *r* is a coefficient that denotes the effect of aging. Additionally, the indicator *x*_*i*_^(*k*)^ denotes the presence of risk factor *k* in patient *i*, initial age *τ*_*i*_ denotes the age of patient *i* at time 0, and *d*_*i*_ denotes the individual variability that follows a normal distribution with mean 0 and variance *σ*^2^. Further, *κ* is the interaction coefficient representing “saturation” of the risk, and *T*_*i*_ denotes the “saturation time” when the hazard of patient *i* reaches its maximum.

### Simulating the development of stroke

**Fig 1:**
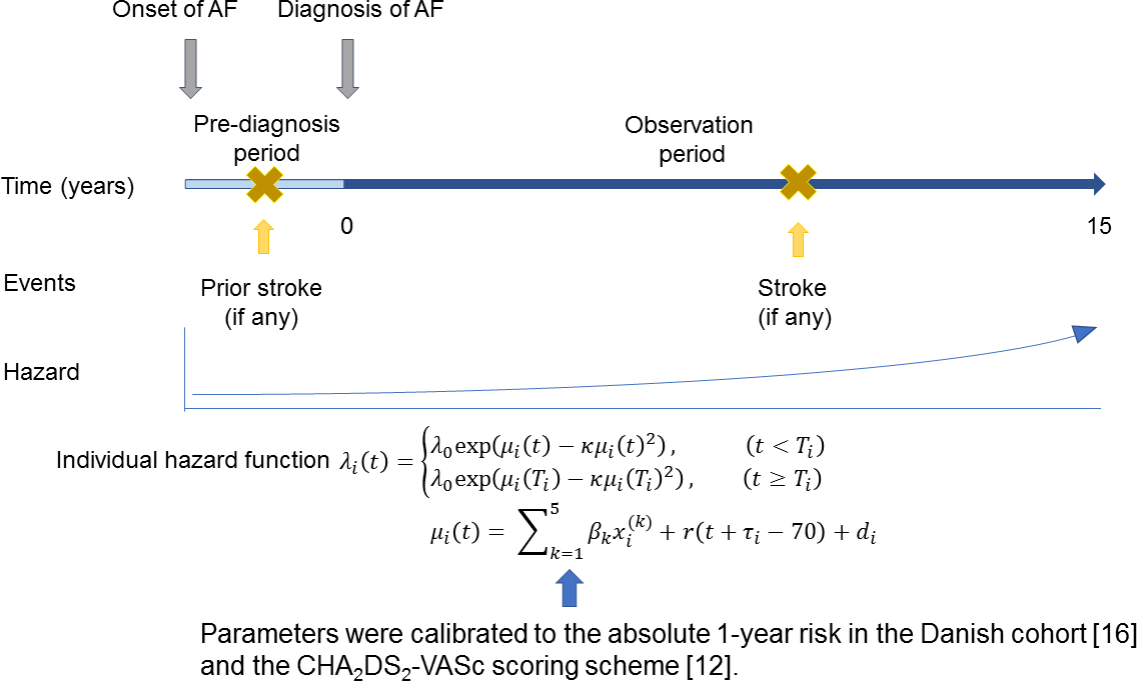
Outline of our stochastic model. The occurrence of stroke was modeled as a nonhomogeneous Poisson process, where the hazard depends on age, comorbidities, and individual variability. If any stroke occurred during the pre-diagnosis period, the period between the onset and diagnosis of AF, it was recorded as a prior stroke. Then, after the diagnosis of AF, the individuals were followed for 15 years or until occurrence of the first stroke. For details, see Methods and S1 Appendix.

We simulated the development of stroke in each individual on the basis of the hazard [22]. We first determined the presence or absence of prior stroke by recording whether the patient suffered a stroke during the pre-diagnosis period, i.e., the period between the onset of AF and its diagnosis. The end of this period, i.e., the time of diagnosis, was defined as the start of the observation period, which extends to 15 years. We then noted the time to onset of the subsequent stroke. Because in real life some patients die from stroke and are thus no longer observed, we introduced a mortality parameter to represent the probability that a patient would be dropped from observation because of stroke occurring during the pre-diagnosis period. With this probability, simulated patients were randomly dropped out at the occurrence of prior stroke. All simulations and statistical analyses were performed with *R* (ver. 3.1.0), a statistical programing environment [23]. Our model is outlined in Fig 1.

### Calculating stroke risk

After computing individual stroke risks from patients’ hazard functions using numerical integration, we calculated the population stroke risk as the mean probability of stroke occurring within the following year for patients with a particular risk profile. We then calculated CHA_2_DS_2_-VASc score-wise stroke risks by taking the mean stroke risks of the populations with matching scores. For this calculation, we excluded TIA from stroke because TIA was excluded from outcomes in the studies we referred to in constructing our model [12,16]. We assumed that TIA comprises 10% of the total events [18, 24], i.e. the hazard for stroke other than TIA was assumed to be 0.9 times the total hazard.

### Setting the parameters

The following population parameters were set: (1) a coefficient denoting the aging effect, (2) coefficients denoting the effects of comorbid risk factors, (3) variance in individual variability, (4) an interaction coefficient denoting saturation, (5) the baseline hazard, and (6) mortality. In addition, the length of the pre-diagnosis period was set.

The parameters constituting the hazard function (parameters 1–5 above) were determined so that the model was concordant with the CHA_2_DS_2_-VASc scoring system [12] and so that the calculated 1-year stroke risks in the simulated populations were in agreement with those observed in one of the largest cohorts ever reported [16]. We chose this reported Danish cohort as a reference cohort not only because of its size but also because it revealed 1-year CHA_2_DS_2_-VASc score-wise stroke risks. The effects of the comorbidities were assumed to be the same and given the same coefficients so that the model would be concordant with the CHA_2_DS_2_-VASc scoring system. The values set are shown in S1 Appendix.

The mortality parameter was set to 0.2, as previously reported [25,26]. The length of the pre-diagnosis period was set to 2 years on the basis of previously reported data [27]. The rationale for and details of the parameter settings are described in S1 Appendix.

### Validating the model

To validate the model, we compared estimated relative risk (RR) for the risk factors in our model against those in the real world. For this purpose, we conducted a “prospective cohort study” in a simulated population. We observed whether stroke developed during the pre-specified “cohort study period,” which immediately followed the diagnosis of AF, and we estimated the hazard ratios (HRs) for age, comorbidities, and prior stroke by means of Cox proportional hazards modeling. The HRs obtained were taken as measures of RR and were compared with RRs reported previously [10,11,19,20]. The cohort size was set to 5 million, and the prevalence of each comorbidity was set to 0.5 so that the widths of all 95% confidence intervals for the estimated HRs were less than 0.1. Initial age was randomly assigned to each patient so that they were between 55 and 85 years of age at the time of diagnosis. The cohort study period was set to 2.5 years so that the mean follow-up time in our cohort study was equal to the median of the mean follow-up times included in the meta-analysis conducted by the Stroke Risk in AF Working Group [10].

### Investigating change in stroke risk along the pFST

To investigate change in stroke risk along the pFST, we simulated 18 populations representing different combinations of age (categorized as <65, 65–75, or ≥75 years) and number of comorbidities (0–5). We further divided each population into two subpopulations, according to the presence or absence of prior stroke, and we simulated development of a subsequent stroke. For patients who remained stroke free for 0, 1, 2, …, 15 years, we calculated the subpopulation-wise mean stroke risk for the year immediately following the pFST. Although initial age was randomly assigned to each patient so that they were between 55 and 85 years of age at the time of diagnosis, because the tendency for stroke to occur in older patients would change the age composition along the FST and could thereby introduce bias, we also investigated the stroke risk in populations for whom we fixed the initial ages at 60, 70, and 80 years rather than generating random ages. The population sizes were determined so that all standard errors of the stroke risk were less than 1.0% of the mean values (S1 Table).

### Investigating the relation between stroke risk and rFST

To investigate the relation between stroke risk and rFST, we simulated 288 populations representing different combinations of age (categorized as <65, 65–75, or ≥75 years), number of comorbidities (0–5), and the candidate rFST (0–15 years). After dividing each population into two subpopulations according to the presence or absence of prior stroke, we simulated the development of stroke during a given candidate rFST. For patients who remained stroke-free during that period, we calculated the subpopulation-wise mean stroke risk for the year immediately following the rFST. Initial age was randomly assigned to each patient so that the patients were between 55 and 85 years of age at the time of diagnosis. We also investigated the stroke risk in populations for whom we fixed the initial ages at 60, 70, and 80 years. The population sizes were determined so that all standard errors of the stroke risk were less than 1.0% of the mean value (S1 Table).

### Viewing stroke risk within the framework of the CHA_2_DS_2_-VASc scoring system: Adjusted CHA_2_DS_2_-VASc score

To examine the change in stroke risk by FST within the framework of CHA_2_DS_2_-VASc scoring system, we defined a new variable, the adjusted CHA_2_DS_2_-VASc score (adjCVS). The adjCVS is calculated on the basis of the proportional change in CHA_2_DS_2_-VASc-wise stroke risk conferred by FST in relation to the patient’s initial CHA_2_DS_2_-VASc score (for pFST) or in relation to the CHA_2_DS_2_-VASc score of the patients with an rFST of 0 (for rFST) (for details, see S2 Appendix). So, for example, a patient with an initial CHA_2_DS_2_-VASc score of 4, could be given an adjCVS of 5 or 3 or even 4.5 or 3.5 after a given pFST, depending on the balance between FEE and aging. Likewise, a patient with a CHA_2_DS_2_-VASc score of 4, could be given an adjCVS of 3.5 depending on their rFST. We applied the adjCVS only to patients without a prior stroke, i.e., patients for whom the FEE was strong. Thus, the highest possible adjCVS was 7.

### Sensitivity analyses

Because mortality and length of the pre-diagnosis period may be uncertain, we performed sensitivity analyses by changing these values. For the combinations of an assumed mortality (0.1, 0.2, 0.3, or 0.5) and length of the pre-diagnosis period (1, 2, or 4 years), we compared two characteristic values that meaningfully show the strength of FEE: (1) the increase in the adjCVS after 10 years of pFST in patients with an initial CHA_2_DS_2_-VASc score of 1 and (2) the rFST required to reduce the stroke risk in patients without a prior stroke from a CHA_2_DS_2_-VASc score of 2 to an adjCVS of 1.5. Each value was obtained in the same model with the new set of parameters determined in the same manner as the original set.

In addition, we examined uncertainty regarding the CHA_2_DS_2_-VASc model. Although CHA_2_DS_2_-VASc scores have proven to be clinically useful [12,16,17], the relative importance of prior stroke may not be strictly twice that of other risk factors. Therefore, we performed a sensitivity analysis by changing the relative importance of prior stroke. The parameters were re-calibrated to the hypothetical scenarios where the risk ratio of the risk conferred by prior stroke against that conferred by other risk factors was 1.6, 1.7, 1.8, 1.9, 2.1, 2.2, 2.3, or 2.4, instead of 2. Thus, each of these scenarios represents a situation where the CHA_2_DS_2_-VASc scoring scheme was somewhat violated and the importance of prior stroke was weighed 1.6 … 2.4, rather than 2. For each scenario, we calculated the above-mentioned indicators of the FEE, and we estimated the HR of prior stoke in the simulated cohort study, performed as described above.

## Results

### Development and validation of the model

**Fig 2:**
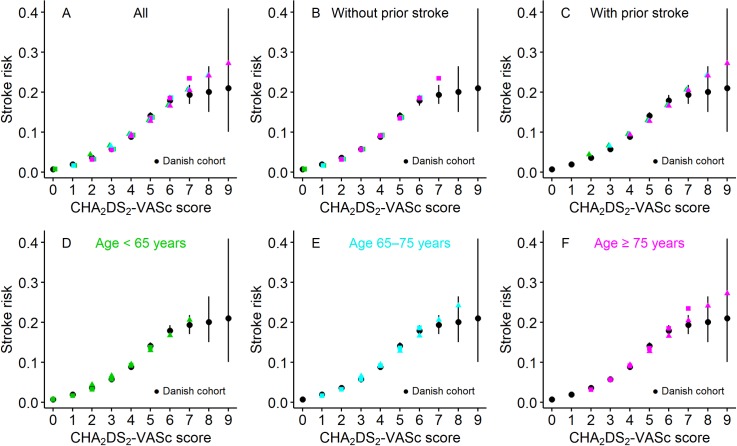
Stroke risk in the simulated populations in comparison to stroke risk in a real-world population. Dot plots of stroke risk in 36 patient populations representing (A) all combinations of risk factors, including 0–5 comorbidities, (B, C) presence or absence of prior stroke, and (D–F) age (categorized as <65, 65–75, or ≥75 years) arranged according to CHA_2_DS_2_-VASc scores. Stroke risk is the probability of stroke occurring within 1 year following the diagnosis of AF. Rectangles (▢) denote absence of prior stroke, and triangles (▵) denote presence of prior stroke. Green denotes age <65 years; blue denotes age 65–75 years; red denotes age ≥75 years. Superimposed are the 1-year stroke risks observed in the Danish cohort [16] with point estimates (dots) and their 95% confidence intervals (error bars).

Calculated risks of stroke within 1 year following the diagnosis of AF were plotted, as shown in Fig 2, for the various combinations of risk factors, i.e., age (categorized as <65, 65–75, or ≥75 years), number of comorbidities (0–5), and presence or absence of prior stroke. The calculated risks were in accordance with those observed in the reference Danish cohort [16] (Fig 2A). They were also in accordance with the CHA_2_DS_2_-VASc scoring system [12]; the calculated risks for patients with the same score were generally in agreement. More precisely, the risks matched when patients had the same total CHA_2_DS_2_-VASc score and the same prior stroke status, regardless of the score components (Fig 2B and 2C). The risks differed slightly when patients had the same total CHA_2_DS_2_-VASc score but a different prior stroke status. The increase in risk conferred by age or comorbidity was slightly greater in patients without a prior stroke than in those with a prior stroke (Fig 2B and 2C). The increase in risk conferred by prior stroke was slightly greater in younger patients with few comorbidities than in older patients with a greater number of comorbidities (Fig 2D–2F). The RRs for age, comorbidities, and prior stroke estimated in the simulated “cohort study” were comparable to those reported in the real world [10,11,19,20], and they largely fell within the 95% confidence intervals from previously reported meta-analyses [10,20] (Table 1).

**Table 1:** Relative risks (RRs) of stroke by aging, comorbidities, and prior stroke in our model and from previously reported meta-analyses.

Risk factor	RR in our model	Previously reported RR
Age (per decade increase)	1.5	1.5 (1.3–1.7)*
Comorbidity		
Hypertension	1.5	2.0 (1.6–2.5)*
Diabetes mellitus	1.5	1.7 (1.4–2.0)*
Congestive heart failure	1.5	NA
Vascular disease	1.5	NA
Female sex	1.5	1.3 (1.2–1.5)^†^
Prior stroke	2.4	2.5 (1.8–3.5)*

*determined by a Stroke Risk in Atrial Fibrillation Working Group meta-analysis of 7 studies [10].

^†^determined by a meta-analysis of 17 studies by Wagstaff et al. [20].

RR or RR (95% confidence interval) is shown.

Abbreviation: NA, not assessed.

### pFST and stroke risk

**Fig 3:**
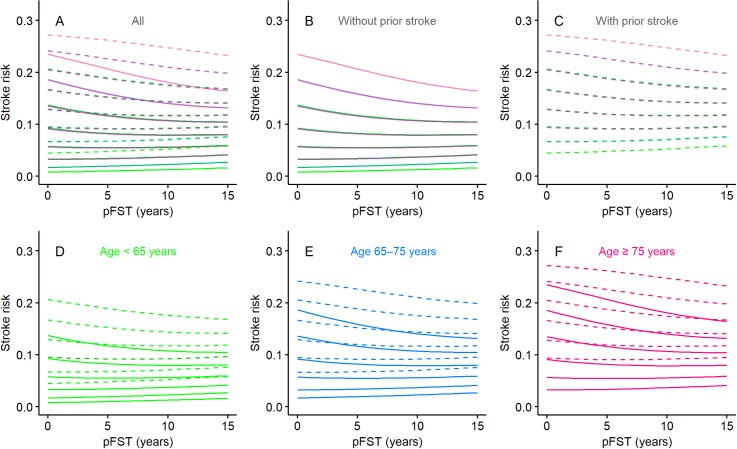
Stroke risks along the pFST. Line graphs showing stroke risk along a pFST of up to 15 years in patient populations representing (A) all combinations of risk factors, including 0–5 comorbidities, (B, C) presence or absence of prior stroke, and (D–F) age (categorized as <65, 65–75, or ≥75 years). Stroke risk is the probability of stroke occurring during the following year. Solid lines denote absence of prior stroke, and dashed lines denote presence of prior stroke. Green denotes age <65 years; blue denotes age 65–75 years; red denotes age ≥75 years. Note that 16 common CHA_2_DS_2_-VASc score-wise trajectories resulted.

**Fig 4:**
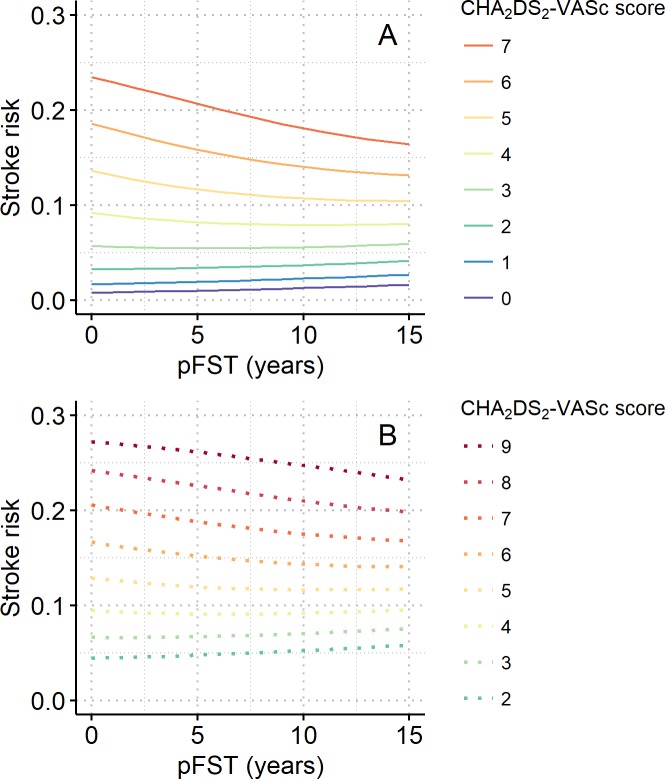
CHA_2_DS_2_-VASc-wise stroke risks along the pFST. Line graphs showing stroke risk along a pFST of up to 15 years per initial CHA_2_DS_2_-VASc-score for patients (A) without prior stroke and (B) with prior stroke. Stroke risk is the probability of stroke occurring during the following year.

Calculated stroke risks were plotted over a pFST of up to 15 years, as shown in Fig 3, for the various combinations of risk factors, as noted above. The stroke risk trajectories overlapped almost completely among patients with matching CHA_2_DS_2_-VASc scores, regardless of their risk factors, provided that the presence or absence of prior stroke matched (Fig 3B and 3C). Generally, the stroke risks did not change much along the pFST, indicating that the FEE and aging offset each other. However, in patients at high risk, the FEE outweighed aging, as shown by the downward trajectories, whereas in patients at low risk, the FEE partially offset aging. The FEE was greater in patients without a prior stroke (Fig 3D–3F, also compare panel B with panel C) and in those with relatively high CHA_2_DS_2_-VASc scores (Fig 3B and 3C). These trends are more clearly depicted in Fig 4, where the CHA_2_DS_2_-VASc score-wise stroke risks are plotted along a pFST of up to 15 years for patients without (panel A) and with (panel B) prior stroke.

**Fig 5:**
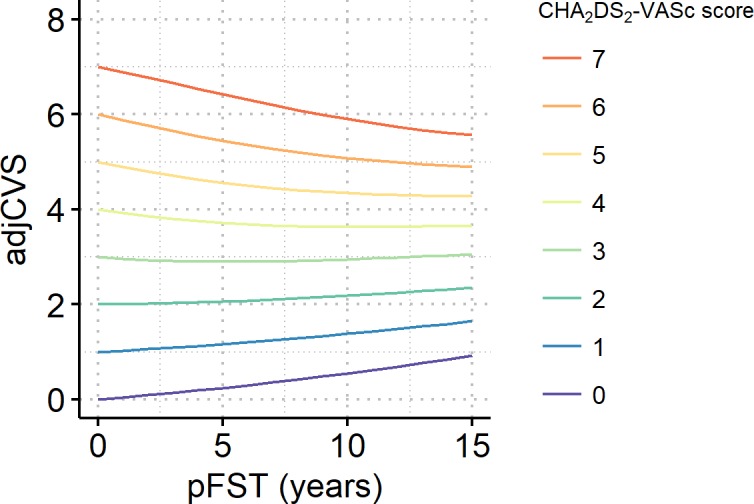
Adjusted CHA_2_DS_2_-VASc scores (adjCVSs) of patients without prior stroke plotted along a 15-year pFST.

For patients without a prior stroke, the adjCVS was plotted along a pFST of up to 15 years (Fig 5). After a pFST of 10 years, the adjCVS increased by 0.55, 0.38, and 0.18 in patients with initial CHA_2_DS_2_-VASc scores of 0, 1, and 2, respectively. Because a 10-year increase in age adds 1 point to the CHA_2_DS_2_-VASc score, the changes in adjCVS indicate that the FEE offset the effect of aging by 45%, 62%, and 82% in patients with initial CHA_2_DS_2_-VASc scores of 0, 1, and 2, respectively. In patients with a CHA_2_DS_2_-VASc score of 3, aging and the FEE offset each other, as shown by an almost constant adjCVS. In patients with a CHA_2_DS_2_-VASc score of 4 or more, the FEE outweighed aging, as shown by the downward slope of the adjCVS trajectories. When patients’ ages were fixed at 60, 70, or 80 years rather than randomly assigned, the adjCVS trajectories were similar (S1 Fig).

### rFST and stroke risk

**Fig 6:**
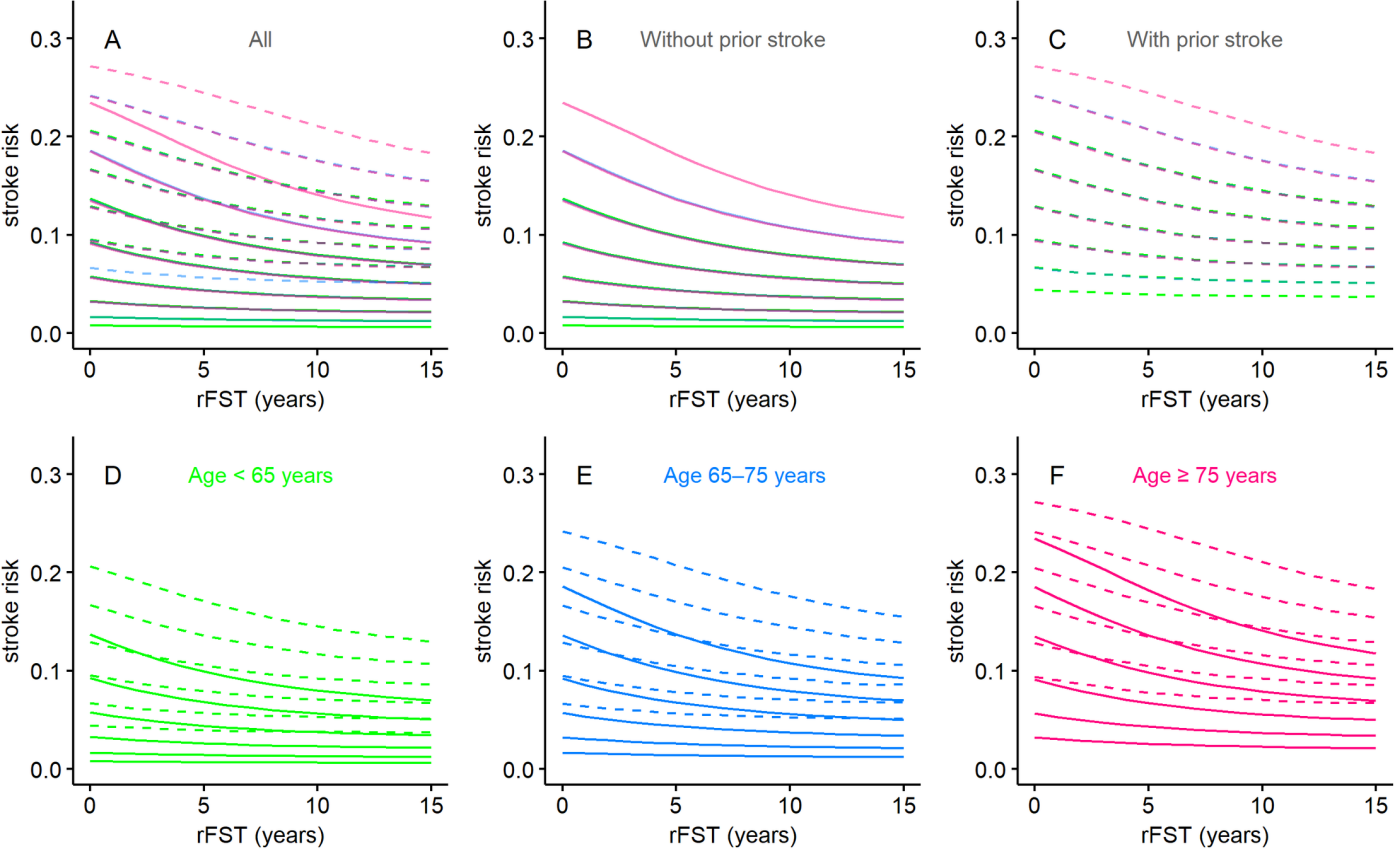
Stroke risks over the rFST. Line graphs showing stroke risk over an rFST of up to 15 years in patient populations representing (A) all combinations of risk factors, including 0–5 comorbidities, (B, C) presence or absence of prior stroke, and (D–F) age (categorized as <65, 65–75, or ≥75 years). Stroke risk is the probability of stroke occurring during the following year. Solid lines denote absence of prior stroke and dotted lines denote presence of prior stroke. Green denotes age <65 years; blue denotes age 65–75 years; red denotes age ≥75 years. Note that 16 common CHA_2_DS_2_-VASc score-wise trajectories resulted.

**Fig 7:**
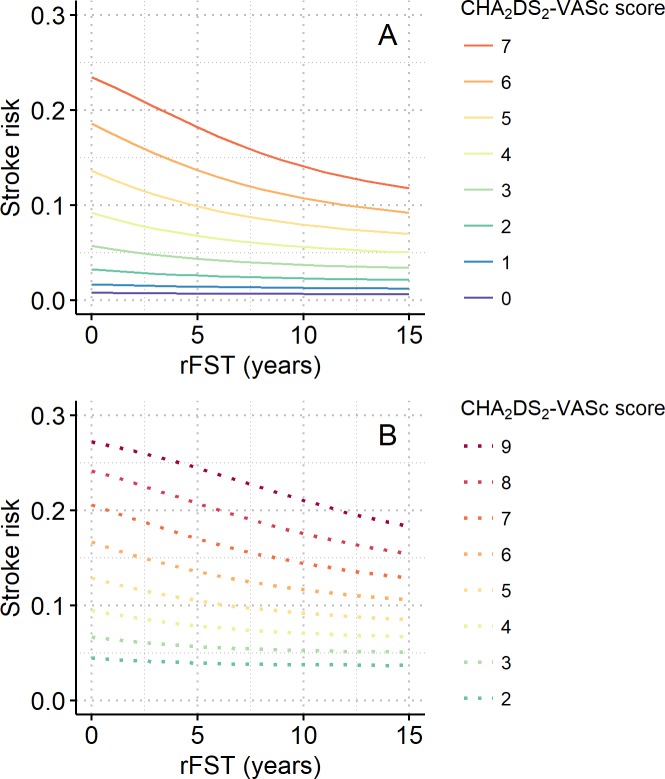
CHA_2_DS_2_-VASc-wise stroke risks over the rFST. Line graphs showing stroke risk over an rFST of up to 15 years per CHA_2_DS_2_-VASc score for patients (A) without prior stroke and (B) with prior stroke. Stroke risk is the probability of stroke occurring during the following year.

Calculated stroke risks of patients with an rFST of 0–15 years were plotted according to the various risk factors (Fig 6). Stroke risk decreased as rFST increased, a trend that reflects the FEE. As was the case for pFST, the trajectories were nearly the same for patients with the same CHA_2_DS_2_-VASc score regardless of their risk factors, provided that the presence or absence of prior stroke matched (Fig 6B and 6C). The FEE was stronger in those without a prior stroke (Fig 6D–6F, also compare panel B with panel C) and in those with a relatively high CHA_2_DS_2_-VASc score (Fig 6B and 6C). These trends are more clearly depicted in Fig 7, which shows the relations between rFST and CHA_2_DS_2_-VASc score-wise stroke risks in patients without (panel A) and with (panel B) a prior stroke.

**Fig 8:**
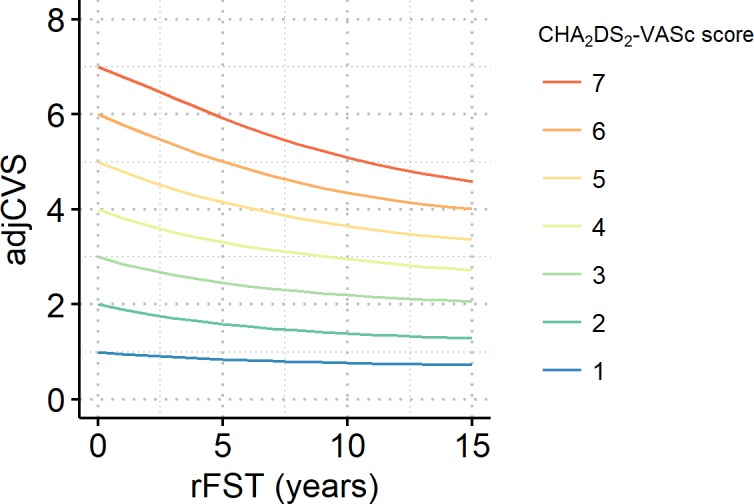
Adjusted CHA_2_DS_2_-VASc scores (adjCVSs) of patients without prior stroke over the rFST. Line graph showing adjCVSs plotted against an rFST of up to 15 years per CHA2DS2-VASc score. For patients with an rFST of 0, the adjCVS is simply the CHA2DS2-VASc score.

For patients without a prior stroke, the relation between rFST and adjCVS is shown in Fig 8. The adjCVS decreased from 2 to 1.5 when the rFST increased from 0 to 6.8 years. This risk reduction by rFST indicates that when patients with a CHA_2_DS_2_-VASc score of 2 have more than 6.8 years of rFST, their “actual score” is nearer to 1, rather than 2, in terms of the present stroke risk. The reductions in adjCVS by 10 years of rFST were 0.24, 0.61, and 0.80 in patients with CHA_2_DS_2_-VASc scores of 1, 2, and 3, respectively. When patients’ ages were fixed to representative ages (60, 70, and 80 years) rather than randomly assigned, the changes in adjCVS were similar (S2 Fig).

### Sensitivity analyses

**Fig 9:**
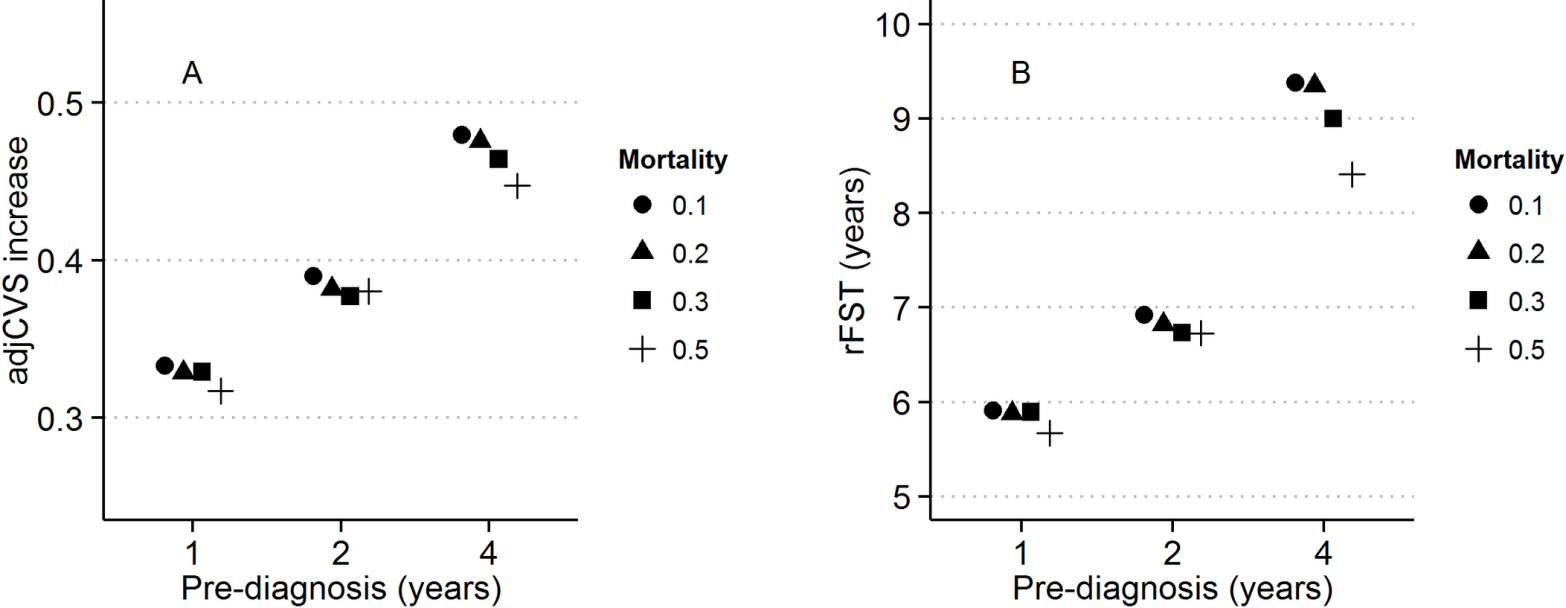
Effects of changes in the mortality parameter and pre-diagnosis period on indicators of the FEE. (A) Increase in adjCVS after 10 years of pFST in patients with a CHA_2_DS_2_-VASc score 1. (B) rFSTs required to reduce stroke risk in patients without prior stroke from CHA_2_DS_2_-VASc score 2 to adjCVS 1.5. Solid circles (●) denote a mortality parameter of 0.1, triangles (▲) denote a mortality parameter of 0.2, squares (■) denote a mortality parameter of 0.3, and plus symbols (+) denote a mortality parameter of 0.5.

Indicators of the strength of the FEE were affected by the length of the pre-diagnosis period; the FEE increased in strength as the pre-diagnosis period decreased in length (Fig 9). The increase in adjCVS after a pFST of 10 years was smaller (minimum, 0.33) or larger (maximum, 0.48), depending on the length of the pre-diagnosis period (1 or 4 years, respectively) (panel A). The rFST required to reduce the stroke risk from a CHA_2_DS_2_-VASc score of 2 to an adjCVS of 1.5 in patients without a prior stroke was shorter (minimum, 5.9 years) or longer (maximum, 9.4 years), depending on the length of the pre-diagnosis period (1 or 4 years, respectively) (panel B). The results were nearly unaffected by the changes in mortality, although the FEE slightly increased in strength as mortality increased.

If the CHA_2_DS_2_-VASc scoring scheme was not exactly satisfied, the FEE was stronger or weaker as the importance of prior stroke as a predictor was made stronger or weaker, respectively, than the CHA_2_DS_2_-VASc score assumes (Table 2). In assuming that prior stroke was only 1.6 times as strong a predictor as other risk factors, the increase in adjCVS after a pFST of 10 years was 0.56, and the rFST required to reduce the stroke risk from a CHA_2_DS_2_-VASc score of 2 to an adjCVS of 1.5 in patients without a prior stroke was 13.3 years. In this situation, the HR of prior stroke was 2.0. At the other extreme, in assuming that prior stroke was as much as 2.4 times as strong a predictor, the increase in adjCVS after a pFST of 10 years was 0.23, and the rFST required to reduce the stroke risk from a CHA_2_DS_2_-VASc score of 2 to an adjCVS of 1.5 in patients without a prior stroke was 4.5 years. In this situation, the HR of prior stroke was 2.8.

**Table 2:** Effect of violation of the CHA_2_DS_2_-VASc scoring scheme on the FEE.

Relative importance of prior stroke specified in the model	HR of prior stroke estimated in the simulated cohort study.	Increase in adjCVS from 1 point after 10 years of pFST	rFST (years) required to reduce the stroke risk from adjCVS 2 points to 1.5
1.6	2.0	0.56	13.3
1.7	2.1	0.51	10.4
1.8	2.2	0.48	9.2
1.9	2.3	0.43	7.7
2.0	2.4	0.38	6.8
2.1	2.5	0.36	6.5
2.2	2.6	0.31	5.6
2.3	2.7	0.28	5.2
2.4	2.8	0.23	4.5

Abbreviations: HR, hazard ratio; adjCVS, adjusted CHA_2_DS_2_-VASc score; pFST, prospective freedom-from-stroke time; rFST, retrospective freedom-from-stroke time.

## Discussion

### The FEE in patients with AF

We modeled various populations of patients with AF. The populations consisted of individuals of various ages and with various numbers of comorbidities, and we simulated the development of stroke in these populations. By such modeling, we were able to quantitatively evaluate the effect of FST on stroke risk. To the best of our knowledge, this is the first study to investigate the FEE in AF, although such an effect has been theoretically predicted [15] and mentioned as an expert opinion [21]. The FEE here is not an individual-level phenomenon as we did not model any temporal change except for aging in individual stroke risk. Rather, the FEE is a population-level phenomenon; it originates from the attrition of high-risk individuals over time. The presence of an FEE means that patients with AF are heterogeneous with respect to stroke risk even if their CHA_2_DS_2_-VASc scores match. It also means that patients who are truly at high risk tend to develop stroke early, whereas those truly at low risk are likely to remain stroke-free. The FEE is a corollary to the fact that prior stroke is a strong predictor of a future stroke [10,11].

### Real-world reproducibility of our model

Our simulation model reproduced important features of the real world: the CHA _2_DS_2_-VASc score-wise stroke risks [16] and the RRs by age, comorbidities, and prior stroke [10,11,19]. Furthermore, because our model respects the CHA_2_DS_2_-VASc scoring scheme [12] when assigning stroke risk, applying our model is equivalent to applying the CHA_2_DS_2_-VASc score model. In addition, our model satisfies proposed criteria for the validation of population-based disease simulation models [14]. Therefore, we believe that the results of our study are clinically relevant.

The only apparent discrepancy between our model and CHA_2_DS_2_-VASc score model is that the risk increase associated with a 1-point increase in age or a comorbidity differed between patients with and without prior stroke. This discrepancy is actually an expected finding. This is because patients with a greater number of risk factors are more likely to have had a prior stroke. Thus, when predicting stroke risk, the presence of prior stroke diminishes the weight of the information contained in the risk factors that are present, and vice versa. Our model has precisely reproduced this expected interaction.

### Relations between the FEE and other risk profiles

According to our study results, the FEE is weaker in patients with a prior stroke than in those without a prior stroke. This means that patients with a prior stroke constitute a relatively homogenous high-risk population, confirming the clinical notion that patients with a prior stroke should be treated as high-risk patients [8,9].

The FEE was strongest in patients with relatively high CHA_2_DS_2_-VASc scores. This trend was an expected finding from a Bayesian perspective [15], and it signifies that once patients with an initially estimated high risk have remained stroke-free, their risk should be re-evaluated a posteriori.

The FEE was strong enough to counterbalance the effect of aging; stroke risk remained largely constant along the pFST. Although we introduced “saturation” into the model, this constancy was not due mainly to the saturation effect; the stroke risk actually increased along with aging when the risk was recorded without regard for the FST (S3 Fig). The offsetting relation between the FEE and aging may explain the apparent paradox observed in some epidemiologic studies—that risk of stroke in patients with AF did not increase as they aged [16,17,28,29]. Interestingly, when only patients with lone AF, patients in whom the FEE is expected to be weak, were followed, the hazard for stroke increased exponentially with age [30]. Although methodological difficulties have precluded epidemiologic evaluation, the FEE in AF may have manifested as such an apparent paradox.

### Clinical implications regarding the decision to initiate OAC

Whether FEE affects the decision to initiate OAC depends on whether it reduces stroke risk below threshold for OAC, which is presumed to be a CHA_2_DS_2_-VASc score of 2 or 1 [8,9]. Because our model includes systemic and pulmonary embolism as outcomes, the threshold may have to be 2 points [18].

In patients with prior stroke, FEE probably does not affect the decision to initiate OAC because their FEE were not strong enough to reduce stroke risk below the threshold; nor in patients with a CHA_2_DS_2_-VASc score of 3 or more, irrespective of the presence or absence of prior stroke.

In contrast, FEE can affect the decision in patients without prior stroke and with a CHA_2_DS_2_-VASc score of 2 or less. In this study, we have found that in simulated patients without prior stroke, a CHA_2_DS_2_-VASc score of 2 practically reduced to less than 1.5 if rFST was more than 6.8 years. This risk reduction by FST suggests that such patients must be regarded as having a CHA_2_DS_2_-VASc score of 1, rather than 2, and thus may affect the decision to initiate OAC.

FEE can also affect the decision in the situation in which patients with a CHA_2_DS_2_-VASc score of 1 grow older to, say, age 75 years, without suffering a stroke. In this study, we found that in simulated patients with a CHA_2_DS_2_-VASc score of 1, FEE offset 62% of the risk increase conferred by 10 years of aging. This offsetting suggests that in such patients, physicians must regard the risk increase conferred by aging to be less than half of the traditionally calculated risk conferred by aging, and this may affect the decision to initiate OAC.

This line of reasoning translates as follows: an rFST of 6.8 years can be regarded as −0.5 points toward the total CHA_2_DS_2_-VASc score, and a pFST of 10 years can be regarded as −0.62 points against the 1 point added for an age increase of 10 years. Thus, both pFST and rFST may have to be considered when deciding whether to start OAC, although rFST may be less important because physicians are always forced to make the decision at the time AF is diagnosed.

### Limitations

The main limitation of our study is that it was model-based, not a real-world study. Although we built the model to reproduce important features of the real world, it was simplified in the following ways: (1) the influence of every risk factor was assumed to be the same including each 10-year increase in age, and (2) the aging effect, pre-diagnosis period, mortality, and interaction (saturation effect) were assumed to be homogeneous with regard to time and to individuals. The CHA_2_DS_2_-VASc scoring scheme, to which we calibrated our model, also simplifies risk factors [12] and is not necessarily consistent with the real world, where the influence of 1 point, as part of the total CHA_2_DS_2_-VASc score, may vary, depending on the constituent factor that the 1 point represents [21]. Indeed, our sensitivity analysis suggested that if the CHA_2_DS_2_-VASc scoring scheme is not exactly satisfied, such that the prior stroke is a more or less important predictor than the CHA_2_DS_2_-VASc score assumes, the FEE may be stronger or weaker, respectively, than the results of this study suggest. The rFST equivalent to −0.5 points as part of the CHA_2_DS_2_-VASc score may vary between 4.5 and 13.3 years, and elimination from the 1-point increase to the CHA_2_DS_2_-VASc score by 10 years of pFST may vary between −0.44 and −0.77, depending on the accuracy of the CHA_2_DS_2_-VASc model.

There is also some uncertainty about pre-diagnosis and mortality parameters. The sensitivity analyses suggested that the FEE might be somewhat stronger or weaker if the pre-diagnosis period in the real world is shorter or longer than we estimated.

Another possible limitation is our application of female sex as a risk factor. Although female sex is a controversial risk factor with possible interaction with age [9,18,19], we treated it as an ordinary risk factor to conform our model to CHA_2_DS_2_-VASc scoring scheme. Our model, therefore, did not capture this possible interaction.

### General implications

The FEE as we defined it is analogous to the recently introduced concept of “conditional survival” in cancer populations [13], and the results of this study suggest that this concept can be extended to situations where a prior event is a risk factor for a recurrent event. Thus, there may be a need to consider the FEE in other disease populations.

Finally, this model may be used to simulate clinical studies (S3 Appendix and S4 Fig) and thereby can serve as a useful aid to designing real-world studies. This model may also be applicable to health economics and decision studies.

## Conclusions

We modeled populations of patients with AF and demonstrated by simulation that the FEE may reduce stroke risk meaningfully and thereby affect the decision to start OAC therapy. The FEE should be considered when stroke risk is evaluated in patients with AF. Results of this study also suggests that the FEE may be important in other recurrent diseases in which a prior event is a risk factor for a future event.

## Supporting information

S1 Table Population sizes and maximal standard errors of stroke risk for simulated populations. (DOCX)

S1 Fig Effect of initial age assignment (random vs fixed) on the trajectories of adjCVS along the pFST. adjCVSs are plotted along a pFST of up to 15 years for patients without a prior stroke according to the initial CHA_2_DS_2_-VASc scores. Solid lines show the stroke risk in relation to pFST when patients’ ages were randomly assigned to either 55–65, 65–75, or 75–85 years, whereas dashed lines show the stroke risk in relation to pFST when patients’ ages were fixed to 60, 70, or 80 years.
(TIF)

S2 Fig Effect of initial age assignment (random vs fixed) on the trajectories of adjCVS along the rFST. adjCVSs are plotted against an rFST of up to 15 years for patients without a prior stroke according to the CHA_2_DS_2_-VASc scores. Solid lines show the adjCVS when patients’ ages were randomly assigned to 55–65, 65–75, or 75–85 years, whereas dashed lines denote the adjCVS when patients’ ages were fixed to 60, 70, or 80 years.
(TIF)

S3 Fig Stroke risk plotted along the duration of AF regardless of the FST. Mean yearly stroke risks were plotted according to the initial CHA_2_DS_2_-VASc scores, regardless of the presence or absence of stroke during the observation period. (A) Patients without prior stroke; (B) Patients with prior stroke.
(TIF)

S4 Fig Stroke risk plotted along the pFST, as estimated by a simulated cohort study. Estimated stroke risks are plotted along the pFST per initial CHA_2_DS_2_-VASc scores for the combinations of age category (<65, 65–75, or ≥75 years) and number of comorbidities (0–2). Stroke risk was estimated as the number of strokes that developed within 1 year (A–C) or within 3 years (D–F) after the diagnosis of AF divided by the total person-years at risk. Solid lines represent point estimates; dashed lines, 95% confidence intervals. Green denotes age <65 years; blue denotes age 65–75 years, red denotes age ≥75 years. Black lines represent the “true” stroke risks calculated by the model.
(TIF)

S1 Appendix Setting the parameters. (DOCX)

S2 Appendix Calculating the adjusted CHA_2_DS_2_-VASc score (adjCVS). (DOCX)

S3 Appendix Simulation of a cohort study conducted to investigate changes in stroke risk along a pFST. Feasibility of the epidemiologic evaluation of the FEE was assessed by simulation.
(DOCX)

S4 Appendix R codes for simulation. (DOCX)
