# Supplementary material for: Changes in stroke risk by freedom-from-stroke time in simulated populations with atrial fibrillation: Freedom-from-event effect when event itself is a risk factor
Source: PLoS One. 2018 Mar 12;13(3):e0194307. doi: 10.1371/journal.pone.0194307 (PMC5847231; doi:10.1371/journal.pone.0194307)
Supplement: S1 Table — (DOCX) [file pone.0194307.s001.docx]

**S1 Table. Population sizes and maximal standard errors of stroke risk for simulated populations.**

**Table A. Simulated population sizes in investigating the relation between pFST and stroke risk.**

| Number of comorbidities |  | Age (years) |  |
| --- | --- | --- | --- |
|  | 55–65 | 65–75 | 75–85 |
| 0 | 3768000 | 2434000 | 1916000 |
| 1 | 2382000 | 1836000 | 1726000 |
| 2 | 1836000 | 1962000 | 2224000 |
| 3 | 1602000 | 2300000 | 3126000 |
| 4 | 2086000 | 2860000 | 4548000 |
| 5 | 3848000 | 6668000 | 8698000 |

**Table B. Maximal standard errors of stroke risk for simulated populations in investigating the relation between pFST and stroke risk.**

| Number of comorbidities |  | Age (years) |  |
| --- | --- | --- | --- |
|  | 55–65 | 65–75 | 75–85 |
| 0 | 0.88% | 0.91% | 0.75% |
| 1 | 0.76% | 0.74% | 0.52% |
| 2 | 0.78% | 0.59% | 0.56% |
| 3 | 0.60% | 0.53% | 0.39% |
| 4 | 0.58% | 0.44% | 0.41% |
| 5 | 0.45% | 0.34% | 0.28% |

Errors are expressed as % of the mean values.

**Table C. Simulated population sizes in investigating the relation between rFST and stroke risk.**

| Number of comorbidities |  | Age (years) |  |
| --- | --- | --- | --- |
|  | 55–65 | 65–75 | 75–85 |
| 0 | 10338000 | 4978000 | 2810000 |
| 1 | 5040000 | 2976000 | 2054000 |
| 2 | 2900000 | 1866000 | 1804000 |
| 3 | 2152000 | 1682000 | 2426000 |
| 4 | 1780000 | 2288000 | 4002000 |
| 5 | 2236000 | 4304000 | 7478000 |

**Table D. Maximal standard errors of stroke risk for simulated populations in investigating the relation between rFST and stroke risk.**

| Number of comorbidities |  | Age (years) |  |
| --- | --- | --- | --- |
|  | 55–65 | 65–75 | 75–85 |
| 0 | 0.64% | 0.51% | 0.74% |
| 1 | 0.74% | 0.64% | 0.57% |
| 2 | 0.49% | 0.50% | 0.39% |
| 3 | 0.66% | 0.43% | 0.58% |
| 4 | 0.39% | 0.46% | 0.37% |
| 5 | 0.34% | 0.29% | 0.27% |

Errors are expressed as % of the mean values.
