# Supplementary figures and images for: Changes in stroke risk by freedom-from-stroke time in simulated populations with atrial fibrillation: Freedom-from-event effect when event itself is a risk factor
Source: PLoS One. 2018 Mar 12;13(3):e0194307. doi: 10.1371/journal.pone.0194307 (PMC5847231; doi:10.1371/journal.pone.0194307)

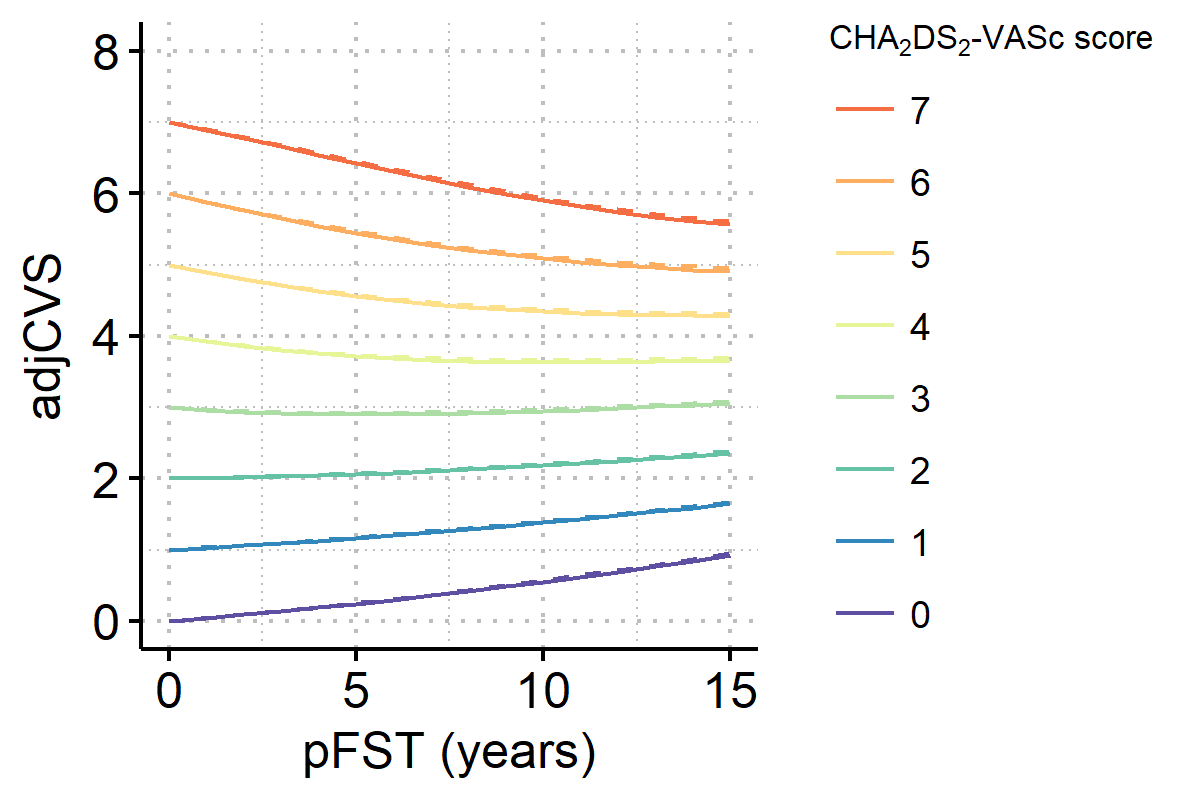

Supplement: S1 Fig — adjCVSs are plotted along a pFST of up to 15 years for patients without a prior stroke according to the initial CHA2DS2-VASc scores. Solid lines show the stroke risk in relation to pFST when patients’ ages were randomly assigned to either 55–65, 65–75, or 75–85 years, whereas dashed lines show the stroke risk in relation to pFST when patients’ ages were fixed to 60, 70, or 80 years. (TIF) [file pone.0194307.s002.tif]

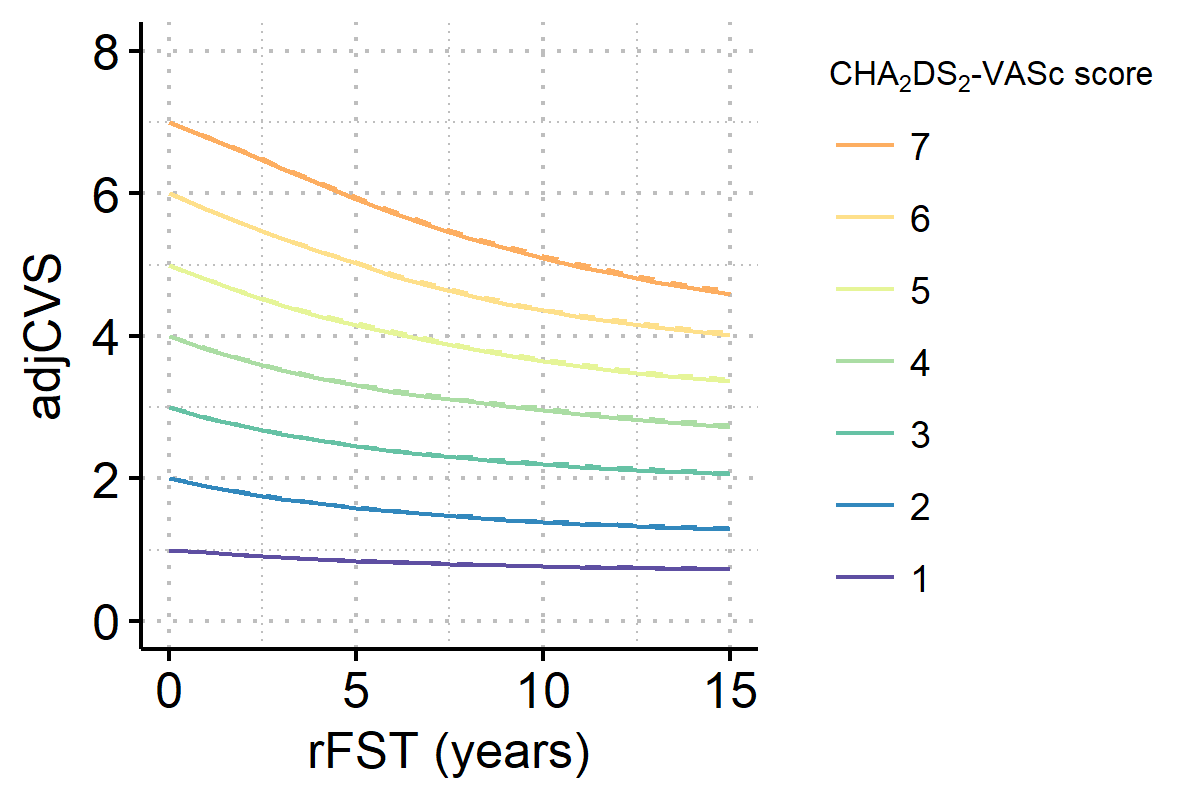

Supplement: S2 Fig — adjCVSs are plotted against an rFST of up to 15 years for patients without a prior stroke according to the CHA2DS2-VASc scores. Solid lines show the adjCVS when patients’ ages were randomly assigned to 55–65, 65–75, or 75–85 years, whereas dashed lines denote the adjCVS when patients’ ages were fixed to 60, 70, or 80 years. (TIF) [file pone.0194307.s003.tif]

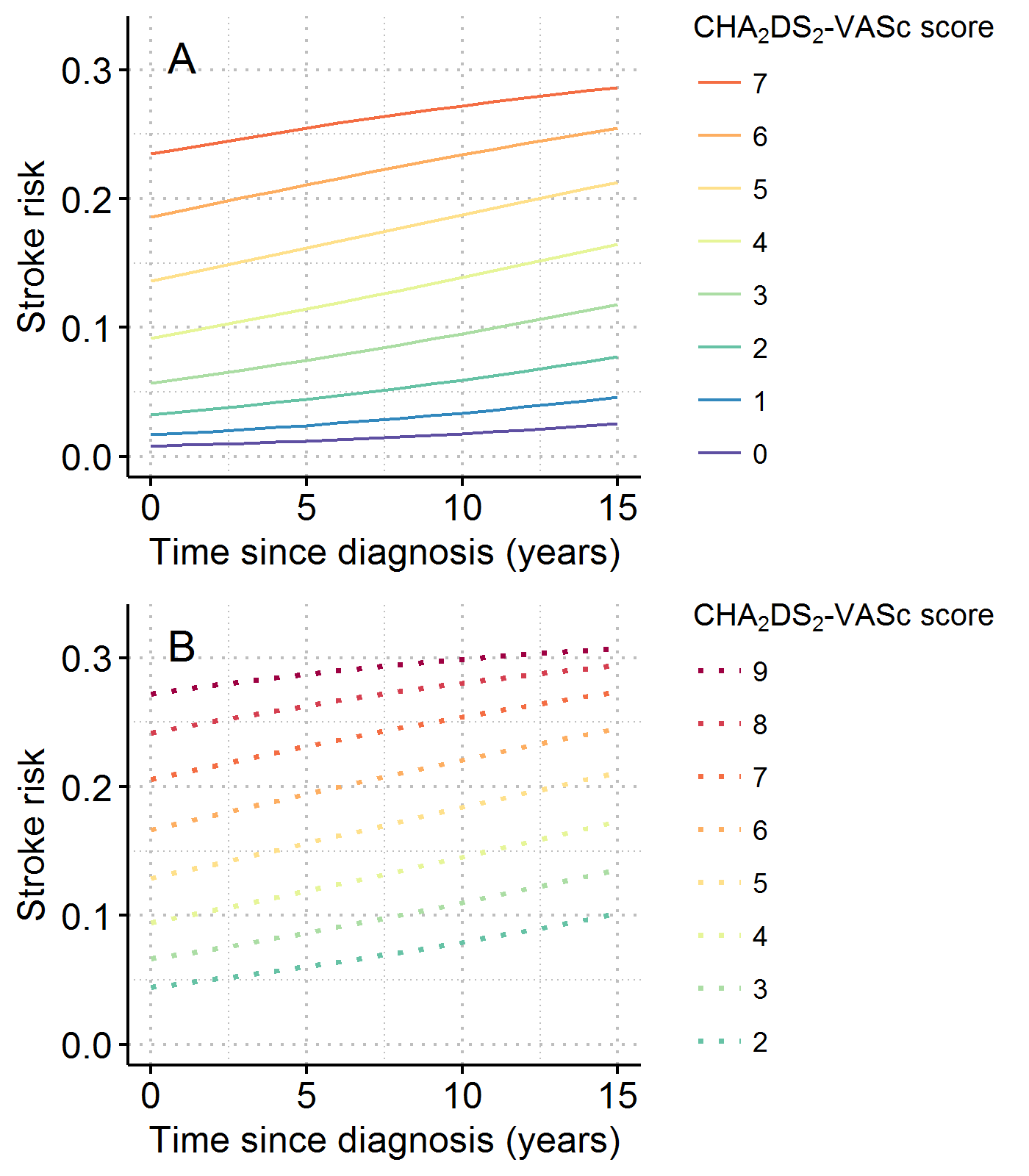

Supplement: S3 Fig — Mean yearly stroke risks were plotted according to the initial CHA2DS2-VASc scores, regardless of the presence or absence of stroke during the observation period. (A) Patients without prior stroke; (B) Patients with prior stroke. (TIF) [file pone.0194307.s004.tif]

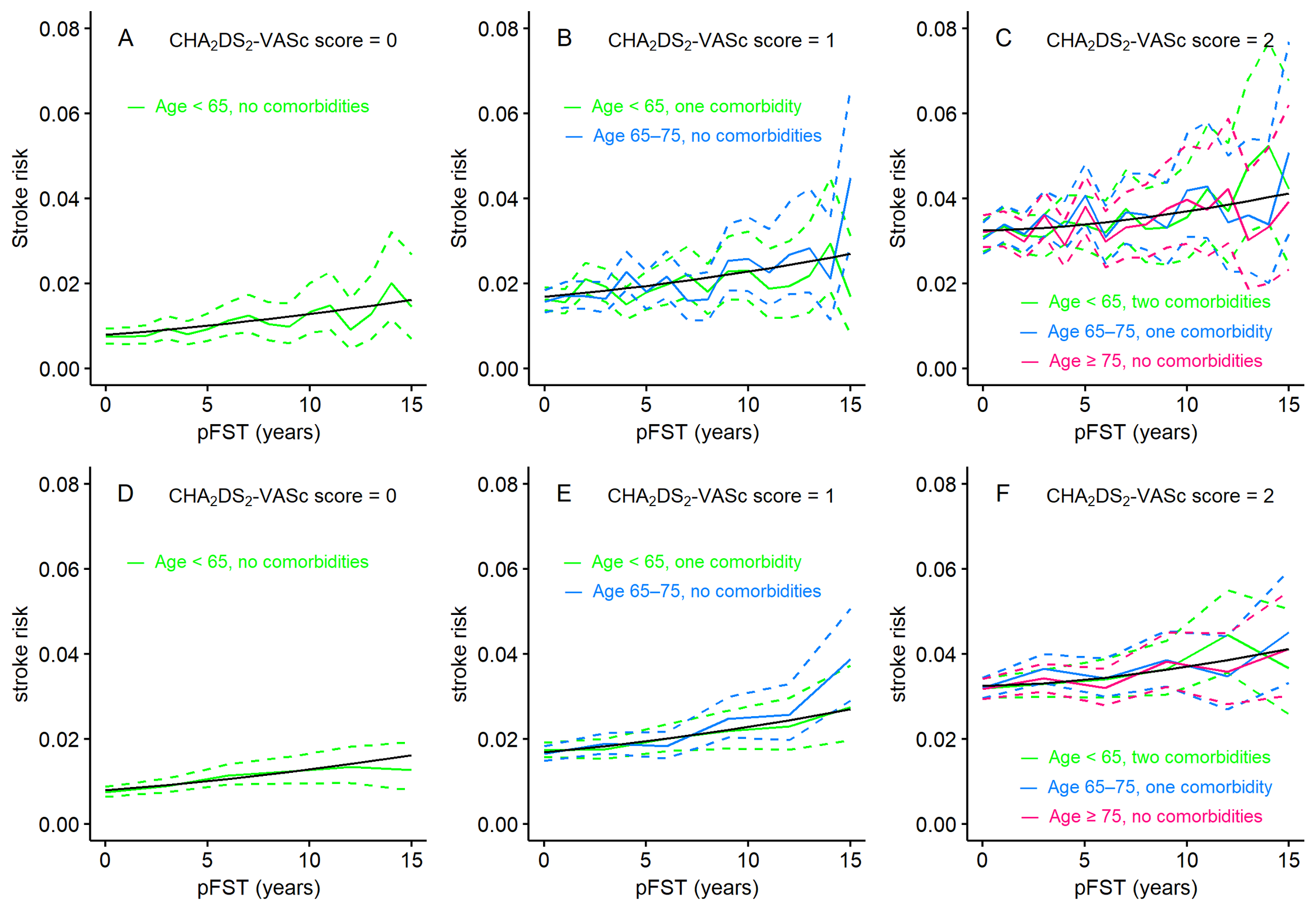

Supplement: S4 Fig — Estimated stroke risks are plotted along the pFST per initial CHA2DS2-VASc scores for the combinations of age category (<65, 65–75, or ≥75 years) and number of comorbidities (0–2). Stroke risk was estimated as the number of strokes that developed within 1 year (A–C) or within 3 years (D–F) after the diagnosis of AF divided by the total person-years at risk. Solid lines represent point estimates; dashed lines, 95% confidence intervals. Green denotes age <65 years; blue denotes age 65–75 years, red denotes age ≥75 years. Black lines represent the “true” stroke risks calculated by the model. (TIF) [file pone.0194307.s005.tif]
