# Supplementary material for: Changes in stroke risk by freedom-from-stroke time in simulated populations with atrial fibrillation: Freedom-from-event effect when event itself is a risk factor
Source: PLoS One. 2018 Mar 12;13(3):e0194307. doi: 10.1371/journal.pone.0194307 (PMC5847231; doi:10.1371/journal.pone.0194307)
Supplement: S1 Appendix — (DOCX) [file pone.0194307.s006.docx]

**S1 Appendix. Setting the parameters**

**Table. Parameter values**

| Parameter | Value |
| --- | --- |
| Baseline hazard *λ*_0_ | 0.007200 |
| Coefficient of aging *r* | 0.1070879 |
| Coefficient of comorbid risk factors *β* | 1.0917875 |
| Variance of individual variability *σ*^2^ | 3.4201754 |
| Coefficient of interaction *κ* | 0.0612590 |
| Mortality | 0.2 |
| Pre-diagnosis period (years) | 2 |

**Setting the parameters (1): hazard function**

The parameters constituting the hazard function were determined so that the calculated stroke risks were in agreement with the real world and so that the model reflected the CHA_2_DS_2_-VASc scoring scheme. For this purpose, the parameters were determined to meet the following two conditions:

1. The 1-year CHA_2_DS_2_-VASc score-wise stroke risks of the simulated populations are consistent with those observed in the reference Danish cohort [17]. This condition was mathematically defined as the minimization of the square sum of the differences in logit-transformed stroke risks weighted by the inverse of the variance.

2. The risk ratios (RRs) for age, comorbidities, and prior stroke are in accordance with their assigned points in the CHA_2_DS_2_-VASc scoring scheme [12], i.e. the ratio of the RRs of age to comorbidities to prior stroke is 1:1:2, as described below.

The parameters were optimized by iterative simulations to realize the minimization condition 1 under the constraint condition 2. The simulations were performed on 18 populations representing separate combinations of age categories in the CHA_2_DS_2_-VASc score (<65, 65–75, ≥75 years) and the number of comorbidities (0–5). We further divided each population into two subpopulations depending on the presence/absence of prior stroke, and we calculated the mean 1-year stroke risk in each subpopulation. Patients’ ages were assigned randomly between 55–65, 65–75, and 75–85 years. The size of each population was determined so that the standard error of the estimated risk of each subpopulation was less than 1.0% of the mean.

In establishing condition 2, the notation *p*(*i*, *j*, *k*) was introduced to denote subpopulation-wise stroke risks, where the index *i* (*i*= 0, 1, 2) represents the age category (<65, 65–75, or ≥75 years), the index *j* (*j*= 0, 1, … , 5) represents the number of comorbidities, and the index *k* (*k*= 0, 1) represents the absence (0) or presence (1) of prior stroke.

Let us examine the first half of condition 2, “RR of age:RR of comorbidity = 1:1.” Put mathematically, we first defined a “*local* age/comorbidity RR ratio” as *p*(*i*+1, *j*, *k*) /*p*(*i*, *j*+1, *k*). Because it can be transformed into {*p*(*i*+1, *j*, *k*) / *p*(*i*, *j*, *k*)}/{*p*(*i*, *j*+1, *k*) / *p*(*i*, *j*, *k*)}, the local ratio represents the ratio of RR of age to that of a comorbidity with a reference risk *p*(*i*, *j*, *k*). Next, we defined a “*global* age/comorbidity RR ratio” as the mean of all local ratios. Condition 2, then, was defined by a global ratio of 1 with < 1% error.

The second half of condition 2, “RR of age and comorbidities:RR of prior stroke = 1:2,” is like that of the first half. The local non-prior/prior ratio was defined as 2*p*(*i*, *j*, 0)/ *p*(*i*, *j*, 1), and the global ratio was defined as the mean of all local ratios.

**Setting the parameters (2): mortality**

The mortality parameter was set to 0.2 because approximately 20% mortality is reported for stroke patients with AF who have not undergone anticoagulation therapy [21, 22]. This value, however, may be an overestimate because our model inherently includes TIA as stroke. To the contrary, it may be an underestimate because patients can go out of observation if they have not died but rather have become severely disabled or placed on OAC after suffering a stroke. Therefore, we also investigated conditions under which mortality was set to 0.1, 0.3, or 0.5.

**Setting the parameters (3): pre-diagnosis period**

Because AF is often clinically silent, it is not possible to pinpoint the onset time in individual patients. Thus, we cannot ascertain the average time between AF onset and diagnosis. We found a clue in the Cardiovascular Health Study [26], where, of 304 AF patients newly identified during the mean follow-up period of 3.28 years, 243 were diagnosed clinically, 36 were diagnosed by annual electrocardiographic screening, and 25 were diagnosed both by annual screening and clinically. On the basis of these data, we estimated the pre-diagnosis period, first by assuming that AF is diagnosed clinically at a constant rate after onset and that AF is detected at annual screenings at a constant probability, which was taken as the proportion of screening-detected cases among those clinically diagnosed. Under these assumptions, we simulated the diagnostic process in patients with AF. In so doing, we found the rate of diagnosis such that, out of the total simulated AF patients newly identified during the 3.28 years, the proportion of patients diagnosed clinically matched the proportion of patients diagnosed clinically in the Cardiovascular Health Study. The pre-diagnosis period was then set as the mean time between the onset and diagnosis of AF among the simulated patients who were likely to have been diagnosed antemortem, i.e., diagnosed within 15 years after onset. This calculation yielded 2.0 years as the pre-diagnosis period. This value, however, is subject to uncertainty. Therefore, we also investigated the conditions that included pre-diagnosis periods of 1 year and 4 years.
