# Supplementary material for: Changes in stroke risk by freedom-from-stroke time in simulated populations with atrial fibrillation: Freedom-from-event effect when event itself is a risk factor
Source: PLoS One. 2018 Mar 12;13(3):e0194307. doi: 10.1371/journal.pone.0194307 (PMC5847231; doi:10.1371/journal.pone.0194307)
Supplement: S2 Appendix — (DOCX) [file pone.0194307.s007.docx]

**S2 Appendix. Calculating the adjusted CHA_2_DS_2_-VASc score (adjCVS)**

The prospective adjCVS is defined as follows, according to the proportional change in stroke risk relative to the patient’s initial CHA_2_DS_2_-VASc score:

adjCVS at the *n*th year = *S*_0_ + (*R_n_* – *R*_0_) /*ΔR,*

where *S*_0_ denotes the CHA_2_DS_2_-VASc score at the time of diagnosis, *R_n_* and *R*_0_ denote the stroke risk at the *n*th year and at diagnosis, respectively, and *ΔR* denotes the difference in initial stroke risk from the nearest upper or lower CHA_2_DS_2_-VASc score.

The retrospective adjCVS is defined similarly:

adjCVS of a patient with a rFST *n* years = *S_n_* + (*R_n_*– *R*_0_) /*ΔR*,

where *S_n_* denotes the CHA_2_DS_2_-VASc score at the time of diagnosis, *R_n_* denotes the stroke risk, and *R*_0_ denotes that of patients with the same score and an rFST of 0, and *ΔR* denotes the difference in stroke risk from the nearest upper or lower CHA_2_DS_2_-VASc score in patients with an rFST of 0.

When the adjCVS changed by 1 or more, it was defined similarly according to the proportional change in stroke risk relative to the nearest CHA_2_DS_2_-VASc score. For example, if the adjCVS for pFST decreases by more than 1 (and no more than 2), the adjCVS is defined as

adjCVS at the *n*th year = *S*_0_ – 1 + (*R_n_* – $R_{0}^{*}$) /*Δ*$R^{*}$*,*

where *R*_0_^*^ denotes the initial stroke risk of the patients with the CHA_2_DS_2_-VASc score *S*_0_ – 1, and *ΔR*^*^ denotes the difference in initial stroke risk between the CHA_2_DS_2_-VASc scores *S*_0_ – 1 and *S*_0_ – 2.
