# Supplementary material for: Changes in stroke risk by freedom-from-stroke time in simulated populations with atrial fibrillation: Freedom-from-event effect when event itself is a risk factor
Source: PLoS One. 2018 Mar 12;13(3):e0194307. doi: 10.1371/journal.pone.0194307 (PMC5847231; doi:10.1371/journal.pone.0194307)
Supplement: S3 Appendix — Feasibility of the epidemiologic evaluation of the FEE was assessed by simulation. (DOCX) [file pone.0194307.s008.docx]

**S3 Appendix. Simulation of a cohort study conducted to investigate changes in stroke risk along the pFST.**

To assess whether the FEE can indeed be studied in the real world, we simulated a cohort study designed to investigate the relation between stroke risk and pFST. The simulation included only patients without a prior stroke and with a CHA_2_DS_2_-VASc score of 0–2 because the FEE is expected to be clinically important in patients such as these. The simulated population comprised 6 subpopulations, each consisting of 10,000 patients and representing different combinations of age category (<65, 65–75, or ≥75 years) and numbers of comorbidities (0–2). The patients were followed for 16 years, and yearly stroke risk was estimated as the number of strokes that developed during the year divided by the total number of the person-years at risk. Censoring was assumed to occur independently at a rate of 15% per year.

Among the total 60,000 patients, 7278 strokes occurred. The total person-years was 301818. Even though this simulated study included a tremendous number of patients, estimation of the stroke risk along the pFST was unstable (Fig S4A–C), and this confirmed the difficulty anticipated in trying to establish the influence of the FEE epidemiologically. However, when we estimated the risk of stroke occurring within 3 years, the estimation was more stable (Fig S4D–F). These simulations suggest that rough epidemiologic evaluation of the FEE is feasible.
