# Supplementary material for: Changes in stroke risk by freedom-from-stroke time in simulated populations with atrial fibrillation: Freedom-from-event effect when event itself is a risk factor
Source: PLoS One. 2018 Mar 12;13(3):e0194307. doi: 10.1371/journal.pone.0194307 (PMC5847231; doi:10.1371/journal.pone.0194307)
Supplement: S4 Appendix — (DOCX) [file pone.0194307.s009.docx]

**S4 appendix. R codes for simulation.**

# **(1) R code for the simulation to investigate the relation between pSFD and stroke risk.**

eventer<-function(lambda0, y, r, I, kappa, mortal){ # I: pre-diagnosis period,

PS<-Death<-t3<-0 # t3: time from Dx to stroke, PS: prior stroke (0: absent 1:present),

# Death: died of prior stroke

hazard<-function(t) {

lambda<-lambda0*exp(y+r*t - kappa*(y+r*t)*(y+r*t))

return(lambda)}

zeta<- -log(runif(1))

Ts<- (1-2*kappa*y)/(2*kappa*r) # time when saturation is reched

K<-lambda0*exp(0.25/kappa) # saturated hazard

if ( Ts < 0 ) { dd<- K*I }

else if (Ts > I) { Risk_next<-integrate(hazard,0,I)

dd <- Risk_next$value}

else { Risk_next<-integrate(hazard,0,Ts)

dd<- Risk_next$value +(I-Ts)*K }

if (zeta< dd) {

PS<-1

while ((zeta < dd) & (Death < 0.5)) {

Death<-rbinom(1,1,mortal)

zeta<-zeta -log(runif(1))

}}

if (Death<0.5){

if (Ts<0) {xi<-zeta/K}

else { L<-integrate(hazard,0,Ts)$value

if (zeta>L) {xi<-Ts + (zeta-L)/K}

else { f<-function(t) {

dd2<-integrate(hazard,0,t)

return(dd2$value-zeta) }

if (f(19.1)<0) {xi<-19.1}

else {x<-uniroot(f,c(0,19.1))

xi<-x$root }

} }

t3<-xi-I }

return(c(t3,PS,Death))

}

riskcal<-function(y1, k, lambda0, r, I, kappa){ #　calculates stroke risk between year k-1 and year k

M1<-length(y1)

lambda_T1<-rep(NA,M1)

for (j in 1:M1) {

Ts<- (1-2*kappa*y1[j])/(2*kappa*r)-I

L<-0.9*lambda0*exp(0.25/kappa) # saturated hazard

hazard<-function(t) {

lambda<-0.9*lambda0*exp(y1[j]+r*(t+I) - kappa*(y1[j]+r*(t+I))*(y1[j]+r*(t+I)))

return(lambda)}

if (Ts <k-1 ) { cumH<- L}

else if (Ts>k) { Risk_next<-integrate(hazard,k-1,k)

cumH<- Risk_next$value}

else { Risk_next<-integrate(hazard,k-1,Ts)

cumH<- Risk_next$value+(k-Ts)*L }

lambda_T1[j]<- 1-exp(-cumH) }

return(mean(lambda_T1))

}

set.seed(100)

N1<-1*matrix(c(3768000,2382000,1836000,1602000,2086000,3848000,2434000,1836000,1962000,2300000,2860000,6668000,1916000,1726000,2224000,3126000,4548000,8698000),6,3)

# size of each population

# To alleviate computational burden, it would be a good idea to replace 1* with a lower value such as 0.2*.

I<-2

mortal<-0.2

parameter<-c(0.007200, 0.1070879, 1.0917875, 1.84937161, 0.0612590)

lambda0<-parameter[1]

r<-parameter[2]

beta0<-parameter[3]

sigma<-parameter[4]

kappa<-parameter[5]

Y2<-15 # maximal observation (years)

# To alleviate computational burden, it would be a good idea to replace 15 with a lower value such as 3.

Y3<-Y2+1

risk_stroke_free0<-risk_stroke_free1<-array(NA,c(6,3,Y3))

# 0: without prior stroke, 1: with prior stroke

for (j in 1:6){

for (k in 1:3) {

t0<-runif(N1[j,k],55,65) # age at Dx

x5<-rnorm(N1[j,k],0,sigma)　 # individual variablility

y<- beta0*(j-1) + x5 + r*(t0+10*(k-1)-70-I)

t3<-PS<-Death<-rep(NA,N1[j,k])

for (i in 1:N1[j,k]) {

EV<-eventer(lambda0,y[i],r,I,kappa,mortal)

t3[i]<-EV[1]

PS[i]<-EV[2]

Death[i]<-EV[3]

}

t3<-t3[Death==0]

y<-y[Death==0]

PS<-PS[Death==0]

t30<-t3[PS==0]

y0<-y[PS==0]

y1<-y[PS==1]

t31<-t3[PS==1]

for (n in 1:Y3) {

yn0<-y0[t30>n-1]

yn1<-y1[t31>n-1]

risk_stroke_free0[j,k,n]<-riskcal(yn0,n,lambda0,r,I,kappa)

risk_stroke_free1[j,k,n]<-riskcal(yn1,n,lambda0,r,I,kappa)

}

} }

R_stroke_free0<-R_stroke_free1<-matrix(NA,Y3,18)

for (k in 1:Y3) {

R_stroke_free0[k,]<-t(as.vector(risk_stroke_free0[,,k]))

R_stroke_free1[k,]<-t(as.vector(risk_stroke_free1[,,k]))

}

par(mfrow=c(1,2)) # simplified graphical presentation

time<-0:Y2

matplot(time,R_stroke_free0[1:Y3,], type="l", ylim=c(0,max(R_stroke_free1)),lty=1,lwd=2,col=rep(c(3,5,6),each=6))

matplot(time,R_stroke_free1[1:Y3,], type="l", ylim=c(0,max(R_stroke_free1)),lty=3,lwd=2,col=rep(c(3,5,6),each=6))

# **(2) R code for the simulation to investigate the relation between rSFD and stroke risk.**

eventer<- function(lambda0,y,r,I,kappa,rFST,mortal){ # I: pre-diagnosis period,

PS0<-PS1<-FALSE # PS0: without prior stroke, PS1: with prior stroke

death<-0 # Death: died of prior stroke

zeta<- -log(runif(1))

haza0<-function(t) {

lambda<-lambda0*exp(y+r*t - kappa*(y+r*t)*(y+r*t))

return(lambda)}

Ts <- (1-2*kappa*y)/(2*kappa*r) # time when saturation is reched

K<-lambda0*exp(0.25/kappa) # saturated hazard

if ( Ts< 0 ) { eta<- K*(I+rFST) }

else if (Ts> I+rFST ) { Risk_next<-integrate(haza0,0,I+rFST)

eta <- Risk_next$value}

else { Risk_next<-integrate(haza0,0,Ts)

eta <- Risk_next$value +(I+rFST-Ts)*K }

if (eta < zeta ) {

PS0<-TRUE }

else {

if ( Ts< 0 ) { dd<- K*I }

else if (Ts> I) { Risk_next<-integrate(haza0,0,I)

dd <- Risk_next$value}

else { Risk_next<-integrate(haza0,0,Ts)

dd<- Risk_next$value +(I-Ts)*K }

while ((zeta < dd) & (death < 0.5)) {

death<-rbinom(1,1,mortal)

zeta<-zeta -log(runif(1))

}

if (zeta >eta) {

PS1<-TRUE }

}

return(c(PS0,PS1,death))

}

riskcal<-function(y1, tau, lambda0, r, I, kappa){

#　calculates stroke risk between year tau and year tau+1

M1<-length(y1)

lambda_T1<-rep(NA,M1)

L<-0.9*lambda0*exp(0.25/kappa)

for (j in 1:M1) {

Ts<- (1-2*kappa*y1[j])/(2*kappa*r)-I

hazard<-function(t) {

lambda<-0.9*lambda0*exp(y1[j]+r*(t+I) - kappa*(y1[j]+r*(t+I))*(y1[j]+r*(t+I)))

return(lambda)}

if (Ts <tau ) { cumH<- L }

else if (Ts>tau+1) { Risk_next<-integrate(hazard,tau,tau+1)

cumH<- Risk_next$value}

else { Risk_next<-integrate(hazard,tau,Ts)

cumH<- Risk_next$value +(tau+1-Ts)* L }

lambda_T1[j]<- 1-exp(-cumH) }

return(mean(lambda_T1))

}

stroke_free<-function(rFST, RFa, T1, T0, N1, r, beta0, lambda0, I, sigma, kappa, mortal){

x5<-rnorm(N1,0,sigma)　 # individual variability

y<- beta0*RFa + x5 + r*(T1 -I + 10*T0-rFST -70)

PS0<-PS1<-rep(FALSE,N1)

death<-rep(NA,N1)

for (i in 1:N1) {

h<-eventer(lambda0,y[i],r,I,kappa,rFST,mortal)

PS0[i]<-h[1]

PS1[i]<-h[2]

death[i]<-h[3]

}

y0<-y[PS0 & (death==0)]

y1<-y[PS1 & (death==0)]

return(c(riskcal(y0, rFST, lambda0, r, I, kappa),riskcal(y1, rFST, lambda0, r, I, kappa)))

}

set.seed(100)

N1<-1*c(10338000,5040000,2900000,2152000,1780000,2236000,4978000,2976000,1866000,

1682000,2288000,4304000,2810000,2054000,1804000,2426000,4002000,7478000)

# size of each population

# To alleviate computational burden, it would be a good idea to replace 1* with a lower value such as 0.2*.

I<-2

mortal<-0.2

parameter<-c(0.007200, 0.1070879, 1.0917875, 1.84937161, 0.0612590)

lambda0<-parameter[1]

r<-parameter[2]

beta0<-parameter[3]

sigma<-parameter[4]

kappa<-parameter[5]

Y2<-15 # maximal observation (years)

# To alleviate computational burden, it would be a good idea to replace 15 with a lower value such as 3.

Y3<-Y2+1

rFST<- 0:Y2 # (candicate) rFST

RF<-rep(0:5,3) # number of commorbidities

T0<-rep(0:2,each=6) # age categories

K1<-length(RF) # number of populations

risk_stroke_free0<-risk_stroke_free1<-matrix(NA,Y3,K1)

# 0: without prior stroke, 1: with prior stroke

for (j in 1:Y3){

for (i in 1:K1) {

T1<-runif(N1[i],55,65)

h<-stroke_free(rFST[j], RF[i], T1, T0[i], N1[i], r, beta0, lambda0, I, sigma, kappa, mortal)

risk_stroke_free0[j,i]<-h[1]

risk_stroke_free1[j,i]<-h[2]

}}

par(mfrow=c(1,2)) # simplified graphical presentation

time<-0:Y2

matplot(time,risk_stroke_free0[1:Y3,], type="l", ylim=c(0,max(risk_stroke_free1)),lty=1,lwd=2,col=rep(c(3,5,6),each=6))

matplot(time,risk_stroke_free1[1:Y3,], type="l", ylim=c(0,max(risk_stroke_free1)),lty=3,lwd=2,col=rep(c(3,5,6),each=6))
